# “Sun Ray” Appearance in a Case of Cardiac Angiosarcoma: A Comparison of MRI and PET/CT

**DOI:** 10.2463/mrms.cr.2015-0082

**Published:** 2016-03-21

**Authors:** Zbyněk Tüdös, Martin Köcher, Marie Černá, František Odstrčil, Zuzana Prouzová, Radim Formánek, Jan Přeček

**Affiliations:** 1 Department of Radiology, Palacky University and University Hospital; 2 Department of Radiological Methods, Palacky University and University Hospital; 3 Department of Clinical and Molecular Pathology, Palacky University and University Hospital; 4 Department of Nuclear Medicine, Palacky University; 5 Department of Internal Medicine I – Cardiology, Palacky University, Faculty of Medicine and Dentistry and University Hospital, I.P. Pavlova 6, Olomouc 77900, Czech Republic

**Keywords:** angiosarcoma, heart, pericardium, MRI, PET/CT

## Abstract

Our article reports a case of a 35-year-old man with cardiac mass, who underwent a wide range of imaging methods, including cardiac magnetic resonance imaging (MRI) and positron emission tomography/computed tomography (PET/CT). Contrast-enhanced MRI images revealed “sun ray” pattern in the mass. Final histopathological diagnosis of angiosarcoma was confirmed during autopsy. To our knowledge, our case is the second direct observation of this MRI diagnostic pattern and the first one that allows a comparison with PET/CT findings.

## Introduction

Primary malignant tumors of the heart are very rare with angiosarcoma being the most frequent one. As with other malignancies, its timely and correct diagnosis is the most important for the prognosis of patients. Unfortunately, despite the technical development and increasing clinical availability of echocardiography, computed tomography (CT), magnetic resonance imaging (MRI), and positron emission tomography/computed tomography (PET/CT), only some of the tumors are detected in the stage allowing curative resection. Modern surgical treatment, chemotherapy and radiotherapy, however, have not reversed the overall poor prognosis of patients with cardiac angiosarcoma. In this paper we present a case study of a patient with angiosarcoma of the heart with emphasis on imaging methods, especially MRI.

## Case Report

**Fig 1.**
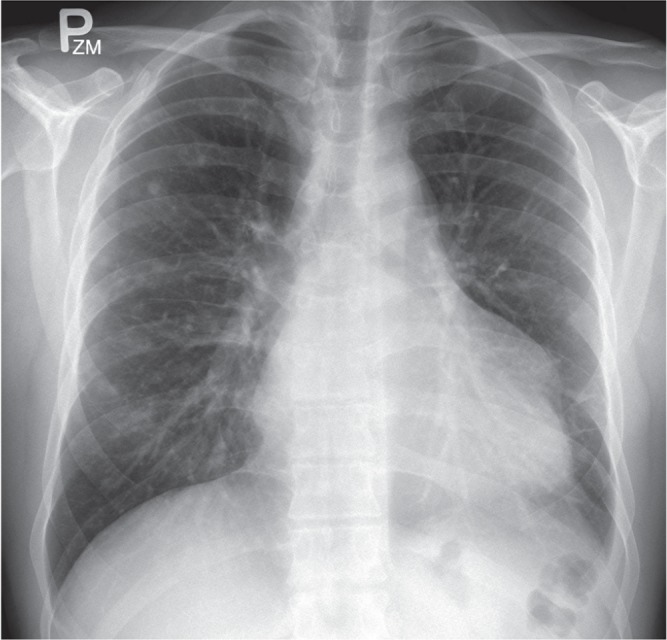
Plain chest x-ray shows abnormal left ventricular contour with silhouette sign. The lung parenchyma contains small pulmonary nodules, later proven to be pulmonary metastases. Blurred left hemidiaphragm and blunted costophrenic angle suggest pleural effusion.

**Fig 2.**
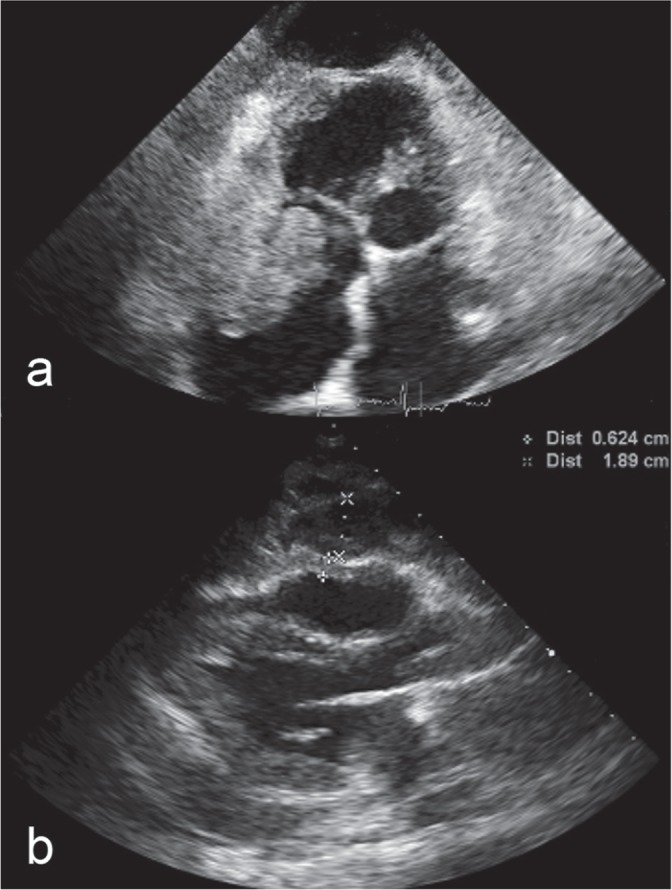
Echocardiography examination revealing (**a**) tumorous mass protruding into the right atrium and (**b**) soft tissue filling the pericardial sac around both ventricles.

A 35-year-old Caucasian male came to the hospital to seek medical care because of abdominal pain, mild dyspnea, and cough. He was examined in the emergency room and a chest x-ray and an abdominal ultrasound were performed. The chest x-ray revealed double contour and enlargement of the left ventricle with silhouette-sign (Fig. 1). Small pulmonary nodules were also detected. Ultrasound demonstrated hepatomegaly. Patient was admitted to the internal medicine department and underwent transthoracic echocardiography, which revealed cauliflower formation protruding from right atrial free wall into the right atrium and also diffuse obliteration of pericardial cavity by pathologic tissue that was surrounding both ventricles (Fig. 2).

**Fig 3.**
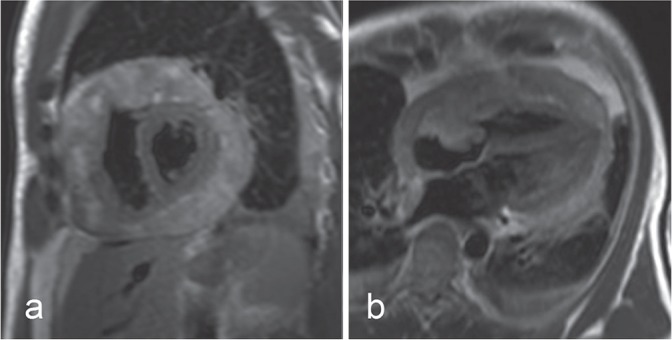
T_2_-weighted “dark blood” turbo spin echo images in (a) short axis and (b) four-chamber plane confirm echocardiographic findings of tumorous mass protruding into the right atrium and soft tissue filling the pericardial sac around both ventricles.

**Fig 4.**
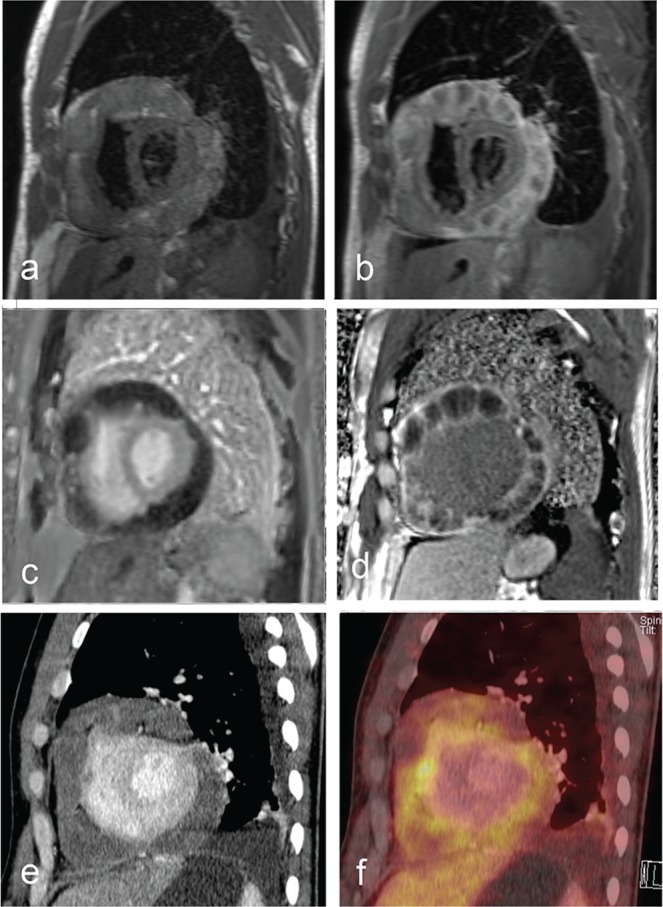
Comparison of different imaging techniques in cardiac short axis plane. (**a**) Non-contrast-enhanced T_1_-weighted (T_1_-w) “dark blood” TSE, (**b**) contrast-enhanced T_1_-w “dark blood” TSE, (**c**) early-enhancement inversion-recovery SSFP, (**d**) late-enhancement inversion-recovery SSFP, (**e**) contrast-enhanced CT, and (**f**) contrast-enhanced CT and PET fusion. “Sun ray appearance” can be recognized on part (**b**) and (**d**), other techniques have failed to display this diagnostic pattern. CT, computed tomography; SSFP, steady-state free precession; PET, positron emission tomography; TSE, turbo spin echo.

Cardiac MRI was performed to further characterize the finding. MRI study used 1.5 Tesla scanner Avanto (Siemens, Erlangen, Germany) and protocol included non-contrast-enhanced electrocardiogram (ECG)-gated balanced steady-state free precession (SSFP) cine sequences [field of view (FOV) 400 ×325 mm, matrix 192 × 156, slice thickness 6 mm, repetition time (TR)/echo time (TE) 41/1.2 ms, flip angle (FA) 54°], ECG-triggered T_2_-weighted double inversion “dark blood” turbo spin echo (T_2_-w TSE) images (FOV 388×337 mm, matrix 384 × 244, slice thickness 8 mm, TR/TE 1300/75 ms, FA 180°) and T_1_-weighted double inversion “dark blood” TSE (T_1_-w TSE) images (FOV 400 × 325 mm, matrix 256 × 125, slice thickness 5 mm, TR/TE 650/32 ms, FA 180°) in standard cardiac planes. Immediately after administration of Gd-DOTA 0.2 ml/kg, we performed T_1_-w TSE images with the same setting as mentioned above, T_1_-w TSE with fat saturation (FOV 340 × 340 mm, matrix 384 × 384, slice thickness 8 mm, TR/TE 641/7.9 ms, FA 180°) and early-enhancement single-shot inversion-recovery steady-state free precession (SS-IR-SSFP) (FOV 444 × 324 mm, matrix 192 × 123, slice thickness 8 mm, TR/TE 570/1.2 ms, FA 40°, inversion time 225 ms). Late-enhancement images using inversion-recovery steady-state free precession (IR-SSFP) (FOV 444 × 402 mm, matrix 256 × 167, slice thickness 8 mm, TR/TE 557/1.3 ms, FA 45°, inversion time was adjusted to null the signal of normal myocardium according to “inversion time scout”) were acquired after 10 minutes. Late-enhancement magnitude and phase sensitive inversion recovery (PSIR) images are part of the routine scanner protocol. In accordance with echocardiography, MRI confirmed a cauliflower mass protruding to the right atrium with extension toward right ventricle, right appendage, and diffuse obliteration of pericardial cavity around the ventricles (Fig. 3). In cine sequences, right ventricle motion was severely impaired by the surrounding and infiltrating mass, flattening and paradoxical motion of interventricular septum was present as a sign of incipient tamponade. Left ventricle systolic function was preserved, but relaxation was also impaired by a mass. Signality of tumor tissue in T_1_-w and T_2_-w TSE was inhomogeneous, mostly isointense to slightly hyperintense (Figs. 3, 4). Pattern of enhancement in T_1_-w TSE and late-enhancement IR-SSFP was also inhomogeneous with lager regions of enhancement in the right ventricular and atrial wall, surprisingly, only thin enhancing rim was visible in the cauliflower mass in right atrium. Tissue within the pericardial cavity contained non-enhancing regions separated by enhancing lines radiating from epicardium to pericardium, this pattern of enhancement (previously described as “sun ray appearance”) was seen both on T_1_-w TSE and late-enhancement IR-SSFP images, especially PSIR images (Fig. 4). Additional findings in MRI images included pulmonary nodules, bilateral pleural effusions, enlarged mediastinal lymph nodes, hepatomegaly, and ascites.

Cardiac angiosarcoma was suspected based on demographic data, tumor location, metastatic spread and visible “sun ray” pattern; in the differential diagnosis, we considered primary cardiac lymphoma, because it also often involves right-sided cardiac chambers or pericardium.

**Fig 5.**
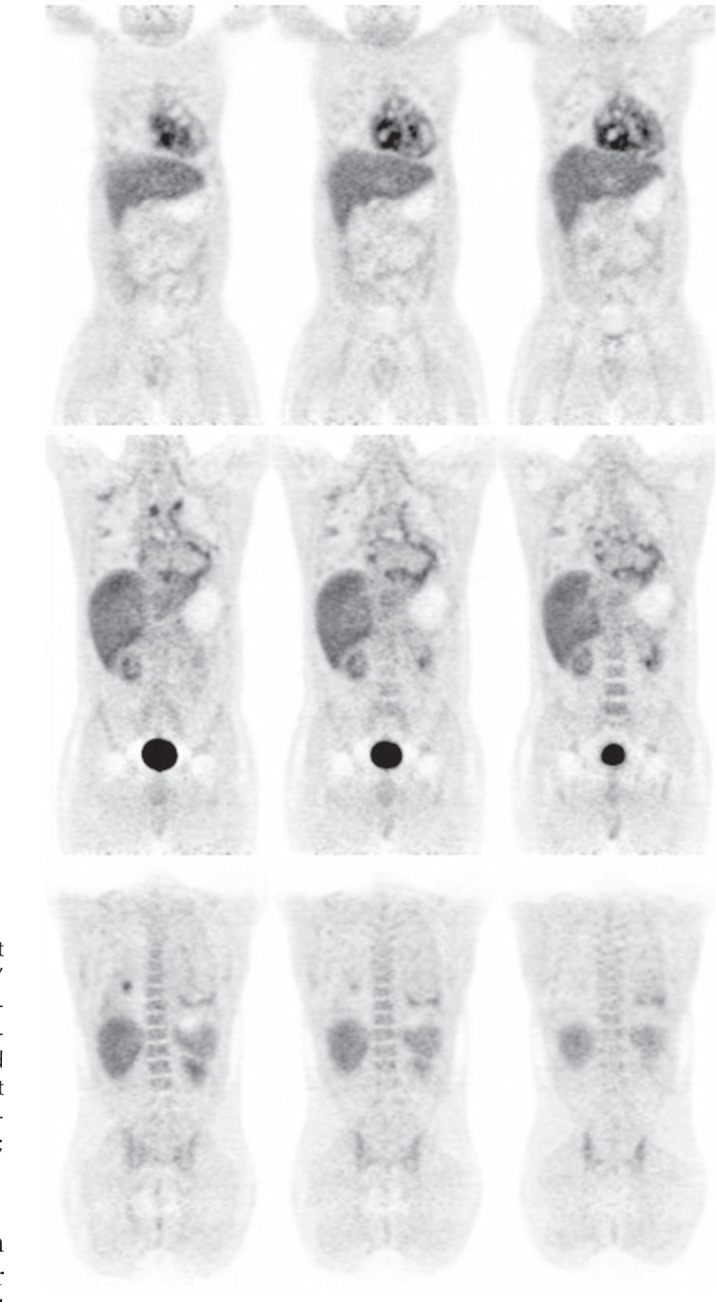
Coronal positron emission tomography images of the torso showing tracer accumulation in heart, pericardium, mediastinum, and in focal lesions in the lungs. These findings were confirmed by clinical autopsy.

Finally, 18-fluoro-2-deoxy-D-glucose PET/CT of the torso was performed using Biograph 16 Hi-Rez scanner (Siemens, Erlangen, Germany), confirming previous morphological findings in the heart and the chest. Regions of increased tracer uptake corresponded to areas of gadolinium enhancement on MRI images. Furthermore, increased tracer uptake was detected in pulmonary nodules and enlarged mediastinal lymph nodes (Fig. 5).

Despite very swift management of diagnostic procedures, clinical status of the patient deteriorated rapidly and he became unable to undergo biopsy. Patient died of right sided heart failure within 1 week after admission.

**Fig 6.**
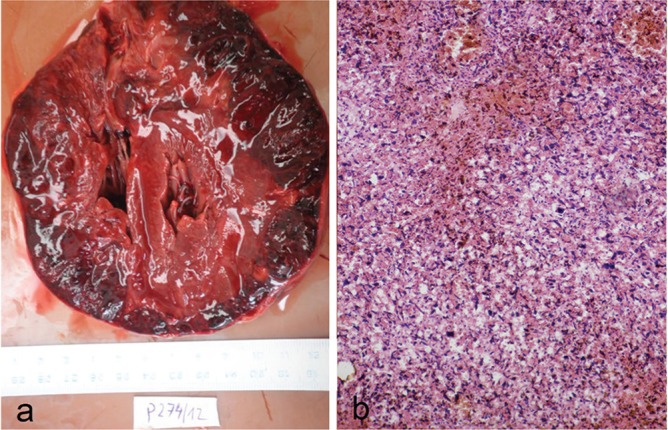
(**a**) Autopsy finding on transverse slice of the heart reveals soft hemorrhagic sponge-like tissue surrounding both ventricles and filling the pericardial sac. (**b**) Hematoxylin and eosin stain (100×) of tumor tissue taken from pericardial sac consisting of organized hematoma with strips of fibrous tissue, clusters of pleomorphic tumor cells, and focal necrosis.

No histology was obtained pre-mortem, thus no specific oncological therapy was applied. Post-mortem autopsy confirmed presence of hemorrhagic sponge-like tissue filling pericardial cavity and adhering to the parietal and visceral layer of pericardium (Fig. 6), and also confirmed the presence of lobulated tumor infiltrating the right atrium free wall and protruding into the right atrium cavity. Autopsy furthermore confirmed the presence of metastases in the lungs, but surprisingly, not in the mediastinal lymph nodes. Clinical autopsy did not discover any other possible primary site of malignancy. Formalin-fixed paraffin-embedded tissue samples were sliced and processed for histological examination. In hematoxylin-eosin stain of tumor part protruding in the right atrium consisted mostly of solid areas of disorganized cells. Tumor cells were highly pleomorphic, mostly spindle-shaped with high mitotic activity and large amount of necrosis. Only minority of the tumor contained poorly differentiated vascular spaces. Hematoxylin-eosin stain of samples taken from pericardial sac consisted of organized hematoma with strips of fibrous tissue, clusters of pleomorphic tumor cells, and focal necrosis. The immunohistochemical profile of tumor cells was positive for CD31, CD34, FVIII and negative for AE1/3 (pan cytokeratins), such a finding is highly specific for angiosarcoma (Fig. 6). The same histological finding was confirmed in lung metastases.

Publication of the case was approved by local ethical committee; informed consent to use patient’s data was waived.

## Discussion

Angiosarcoma is the most common type of primary malignant tumors of the heart.^1^ The tumor occurs more often in men than in women (in ratio around 2:1), the average age of patients is around 40 years.^1^ The most frequent localization of the tumor is the free wall of the right atrium (about 60% of cases) or together with involvement of the right ventricle or the adjacent pericardium (about 20% of cases).^1^ Isolated involvement of the pericardium, the right ventricle, or the left atrium is rather rare (4–6.5% of cases).^1^ Obliteration of pericardial cavity by tumor or fibrous tissue is present in 31–43%.^1^

Unfortunately, diagnosis of angiosarcoma is usually late and metastases are present in 66–89% of cases, typically in the lungs, liver, lymph nodes, or the central nervous system; other possible sites of metastases include bones, kidneys, and the spleen.^1^

Clinical manifestation depends on tumor localization; frequent symptoms include fatigue, malaise, dyspnea, signs of right ventricular failure, and chest or abdominal pain.

Our 35-year-old male with abdominal pain and dyspnea can be considered a typical representative regarding age, gender, and clinical manifestation of angiosarcoma.

Imaging methods play a crucial role in the diagnosis of malignant tumors. A plain chest x-ray might show pathological changes, such as cardiomegaly and abnormality of heart shadow, mediastinal mass, pleural effusion, or signs of dissemination into the lungs. Plain chest x-ray was performed in our patient because of cough and dyspnea and the revealed abnormal left ventricle contour prompted further imaging studies. Transthoracic echocardiography is a readily available method enabling detection of a pathological cardiac mass and evaluation of its relationship to cardiac ventricles, valves, and the pericardium.

Generally, tomographic imaging methods usually display angiosarcoma as irregular lobulated cauliflower formation involving free wall of the right atrium, possibly infiltrating also the surrounding pericardium or the free wall of the right ventricle. In the CT image, the tumor appears as a low-attenuation right atrial mass,^2^ but CT does not allow further tissue characteristics.

An MRI represents an important tool in the evaluation of cardiac tumors, their anatomic definition, tissue characterization, and functional impact.^3^ Recommended MRI protocol should include balanced SSFP cine images in standard cardiac planes, T_1_- and T_2_-weighted double inversion recovery “dark blood” TSE images, spectral presaturation fat suppression images, and gadolinium-enhanced black blood TSE._3_ Contrast-enhanced T_1_-w TSE can be performed immediately after the contrast media administration,^4,5^ or alternatively, T_1_-w spoiled gradient-echo first-pass perfusion^3^ or early-enhancement SS-IR-SSFP^6^ can be performed. It is recommended to add late-enhancement IR-SSFP after 10 minutes.^3,4,6^ Angiosarcoma typically appears iso- to hyperintense in T_1_-w TSE images and hyperintense on T_2_-w TSE.^2,4^ Generally, it could be stated that angiosarcomas are mostly strongly inhomogeneous and their signal characteristics depend on the content of hemorrhage, vessels, or necrosis. After contrast media administration, angiosarcomas typically show strong and irregular or rim enhancement. In 1994 Yahata et al. observed enhancing lines radiating from the epicardium to parietal pericardium and called this type of enhancement “sun ray appearance.”^7^

In our case, we observed an identical MRI finding that was caused by the presence of pathological tissue filling the pericardial sac. To compare the MRI technique used, Yahata et al. observed the pattern using contrast-enhanced T_1_-w TSE, we used the same plus early- and late-enhancement IR-SSFP. The “sun ray” sign was seen on the T_1_-w TSE images (Fig. 4b), but on late-enhancement PSIR-SSFP images it was seen even better (Fig. 4d), on the other hand, the sign could not be distinguished on an SS-IR-SSFP (Fig. 4c). By comparing MRI with the autopsy and microscopic findings, we conclude that hypointense regions on Fig. 4b and d are attributable to unenhancing organized hematoma and necrosis, while hyperintense lines are attributable to enhancing bands of fibrous tissue, with typical persistence of gadolinium in a late-enhanced phase. Interestingly, “sun ray” pattern is often mentioned in reviews and articles about malignant tumors, but it is worth noting that Yahata’s observation has long been the only case described and the second direct observation was published in 2013 by Akkaya et al.^8^ But it is important to stress that Akkaya et al. used the term “sun ray appearance” for MRI findings of signal-void in parallel vessels extending from the epicardium towards the pericardium seen on unenhanced T_2_-w TSE images. So compared to ours and Yahata’s cases, Akkya’s finding was based on a bundle of several macroscopically visible parallel vessels with flowing blood, that were best seen as flow-voids on unenhanced T_2_-w TSE images, whereas our finding was based on regions of organized hematoma, necrosis, and enhanced bands of fibrous tissue best seen on late-enhancement PSIR-SSFP. We did not observe the finding described by Akkya et al. on T_2_-w TSE in our patient (Fig. 3). So despite the fact, that MRI finding led Akkaya et al. to a correct suspicion of angiosarcoma, MRI appearance and the technique used differ a lot from the original Yahata’s article.^7,8^ To our best knowledge, our case report is the second direct observation of a pattern identical to that described by Yahata et al. It can therefore be argued that “sun ray appearance” is either little published, or is extremely rare.

An increasing number of angiosarcomas have been studied with PET/CT. This hybrid method allows evaluation of both morphology and metabolism of tumors and may aid in differentiation between benign and malignant tumors, furthermore it is also very suitable for detection of distant metastases and follow-up of patients after treatment.^9,10^ PET/CT study in our patient showed good correlation of morphological MRI and CT findings regarding involvement of the heart and pericardium, tracer uptake within the cardiac mass was irregular and increased only in several focal regions corresponding to areas of enhancement in MRI images, but neither CT nor PET images displayed clear linear “sun ray” pattern. Compared to echocardiography, CT, or PET, only MRI has sufficient contrast and spatial resolution needed to distinguish the strips of fibrous tissue within a hematoma and necrosis, thus we assume MRI to be the only imaging method able to depict the “sun ray appearance.” The differential diagnosis of cardiac angiosarcoma includes other types of sarcomas (e.g., rhabdomyosarcoma, undifferentiated sarcoma, etc.), as well as lymphoma that often affects the right atrium free wall and pericardium.^2,11^ Differential diagnosis of cardiac angiosarcoma and lymphoma using MRI is addressed in very recent paper comparing series of seven angiosarcomas and five lymphomas.^12^ Authors describe different type of enhancement in the two groups—six out of seven sarcomas revealed enhancing rim with unenhancing necrotic central part, whereas the enhancement of all lymphomas was rather homogeneous. Other possible distinguishing features mentioned in the paper are age (patients with sarcomas were younger), involvement of the right appendage (present in all sarcomas), and extension of tumor toward the right ventricle (present in all lymphomas, but only three sarcomas). Despite advances in imaging techniques and their increasing clinical availability, most patients are diagnosed at an advanced stage and the vast majority of patients die within 12 months since diagnosis.^1^

We can conclude that our patient was a typical case in terms of gender, age, and tumor localization. Unfortunately, he also confirmed a quick spread and generally poor prognosis of cardiac sarcomas. The finding was detected by chest x-ray, confirmed by echocardiography and further specified using cardiac MRI and PET/CT. Suspicion of angiosarcoma was raised based on the tumor localization, the presence of distant metastases, and “sun ray appearance” on the contrast-enhanced MRI images.
